# Quantified assessment of hyperactivity in ADHD youth using IR-UWB radar

**DOI:** 10.1038/s41598-021-89024-7

**Published:** 2021-05-05

**Authors:** Won Hyuk Lee, Johanna Inhyang Kim, Amy M. Kwon, Jong Ho Cha, Daehyeon Yim, Young-Hyo Lim, Seok-Hyun Cho, Sung Ho Cho, Hyun-Kyung Park

**Affiliations:** 1 Department of Electronics and Computer Engineering, Hanyang University, 222 Wangsimni-ro, Sungdong-gu, Seoul, 04763 Republic of Korea; 2 Department of Psychiatry, Hanyang University Medical Center, Seoul, Republic of Korea; 3 Biostatistical Consulting and Research Laboratory, Medical Research Collaborating Center, Seoul, Republic of Korea; 4 Division of Neonatology, Department of Pediatrics, Hanyang University College of Medicine, 222 Wangsimni-ro, Seongdong-gu, Seoul, 04763 Republic of Korea; 5 Xandar Kardian Inc, Seoul, Republic of Korea; 6 Division of Cardiology, Department of Internal Medicine, Hanyang University College of Medicine, Seoul, Republic of Korea; 7 Department of Otorhinolaryngology, Hanyang University College of Medicine, Seoul, Republic of Korea

**Keywords:** Biomedical engineering, Translational research

## Abstract

Research on the quantification of hyperactivity in youth with attention-deficit/hyperactivity disorder (ADHD) has been limited and inconsistent. The purpose of this study was to test the discriminative value of impulse-radio ultra-wideband (IR-UWB) radar for monitoring hyperactive individuals with ADHD and healthy controls (HCs). A total of 10 ADHD patients and 15 HCs underwent hyperactivity assessment using IR-UWB radar during a 22-min continuous performance test. We applied functional ANOVA to compare the mean functions of activity level between the 2 groups. We found that the mean function of activity over time was significantly different and that the activity level of the ADHD group slightly increased over time with high dispersion after approximately 7 min, which means that the difference in activity level between the two groups became evident at this period. Further studies with larger sample sizes and longer test times are warranted to investigate the effect of age, sex, and ADHD subtype on activity level function.

## Introduction

Attention-deficit/hyperactivity disorder (ADHD) is a childhood-onset neurodevelopmental disorder that is characterized by three cardinal symptom domains: inattention (IA), impulsivity and hyperactivity. Hyperactivity is considered a core and ubiquitous characteristic of ADHD^1^, and the DSM-5 defines hyperactivity as “frequent fidgeting of hands or feet, difficulties in remaining seated, inappropriate running or climbing, and/or acting as if driven by a motor”. Early recognition of hyperactivity remains a challenge as young children are considered generally active and it is difficult to determine a deviate activity level from the norm^2^. The severity of hyperactivity is regarded to change over the life span, as the gross motor hyperactivity in children is assumed to change towards fidgeting and a more subtle sense of restlessness with increasing age^3^. ADHD children have been known to display hyperactivity in specific contexts, such as academic classes^4^. The activity levels of ADHD children according to different situations over time have not been well studied^5^.

There is no consensus on the best method to quantify and assess activity level. Currently, the determination of excess gross motor activity in clinical settings mostly relies on subjective tools, such as clinicians’ observations, reports by caregivers or questionnaires^6^, but these measurements are inherently limited by informant reporting bias, which can lead to discrepancies in the diagnosis of ADHD^7^. The most frequently used research techniques have been infrared motion analysis and accelerometer-based devices (actigraphy and inertial measurement units (IMUs)), but both methods have produced controversial results.

Several commercial devices incorporating infrared motion tracking cameras with computerized attention evaluations, such as OPTAx, the McLean Motion and Attention Test System (MMAT) and the quantified behavior test (Qb test)^8^, are available. Among these, the most well-studied tool is the Qb test, which was approved by the Food and Drug Administration (FDA) as a diagnostic aid and treatment monitoring system^9^. Head movement is measured with an infrared camera that tracks a reflective marker attached to a headband worn by the participant while undergoing a 20-min continuous performance test (CPT). Despite its good psychometric properties, recent studies have shown that the Qb test cannot differentiate ADHD from other neurodevelopmental disorders^10,11^. Notably, the Qb test would likely fail to detect movements if the participant were to deviate from the test area and is not sensitive to the movements of body parts other than the head.

Accelerometer devices can record fine-grain activity information (i.e., speed and timing of movement) that may be particularly relevant for the assessment of activity^12^. They can also detect movement of the arms or legs if worn on the wrist or ankle. The strength of accelerometer devices lies in their portability, making the assessment of activity in naturalistic setting possible. Despite numerous studies on its clinical utility^1,13^, no gold standard protocol has been established for actigraphic measurements^14^. Time periods of assessment vary widely, and study results vary according to the position of the devices^12^. Moreover, certain movements (such as fidgeting-like activities involving the upper body) are unlikely to be assessed adequately^15^.

Impulse-radio ultra-wideband (IR-UWB) radar combines the advantages of the previous infrared motion tracking devices and accelerometer-based devices, as both the quantification of movements and tracking of spatial movements are possible. Due to the very low power required to transmit and receive signals to comply with Federal Communications Commission (FCC) standards, IR-UWB radar can detect a person's movements continuously in an indoor environment. Our previous study results demonstrated the superiority of IR-UWB radar in detecting changes in spatial position and sedentary micromovements compared to actigraphic sensors^16^. The radar’s ability to detect every change in the environment through electromagnetic waves enables it to capture a wide range of body movements as well as the movement of specific body parts. Based on these characteristics, we suggest that the IR-UWB radar can be a useful tool to objectively evaluate hyperactivity in ADHD patients. Another merit of IR-UWB radar is that it can be applied in various situations, such as during a CPT or in a naturalistic setting.

The purpose of this study was to compare the activity level of youth with ADHD with that of healthy controls (HCs) using four IR-UWB radar sensors during a 22-min CPT. The median value of the signals detected by the four radars during one minute was called the QAR (quantified assessment of hyperactivity using IR-UWB radar) score, and total activity during the 22 min were calculated by the sum of the 22 QAR scores. We compared the total activity level during the 22-min period, and we also assessed the temporal variance in activity by applying functional ANOVA (fANOVA), which is a statistical method used to analyze continuous-time monitoring processes whose final outputs are samples of functions. Functional data is data providing information about curves, surfaces or anything else varying over a continuum. Analysis of longitudinal curve data is challenging, and previous researchers focused on simple summary measures, thereby discarding potentially important information. FANOVA implements interpolation, smoothing and derivation to effectively highlight the characteristics and identify patterns of variation. In fANOVA, we compared the mean function and velocity of total movement between the two groups. The mean function of movement indicates the representative function of the movements from all subjects in a group, which is calculated by the arithmetic mean values at a given time point. The velocity of total movement refers to the rate of change in movement within a group during a given time point.

As previous meta-analyses observed significantly greater activity in individuals with ADHD on both actigraphy and motion tracking data with medium to large effect sizes^6,8^, we hypothesized that (1) the ADHD group would have a higher average activity level than the HC group. Based on results of a previous study showing that hyperactivity levels increases with time^17^, and also results of studies showing that the time-on-task affects task performance in ADHD patients^18^, we hypothesized that (2) the activity level would increase over time in the ADHD group, whereas it would remain relatively stable in the HC group.

## Results

The demographic and clinical characteristics of the two groups are presented in Table 1. There were no significant differences in age, sex or IQ between the two groups. Among the 10 ADHD patients, 8 had the predominantly inattentive subtype, while 2 had the combined subtype. In addition, the sum of the total QAR scores, which reflects the total amount of movement during the 22 min, did not differ significantly between the ADHD group and the HC group (Table 1).

**Table 1:** Demographic information and comprehensive attention test of subjects.

	ADHD (n = 10)	Controls (n = 15)	*p*-value
**Sex**			
Male, N (%)	6 (60.0)	10 (66.67)	0.3087
Female, N (%)	4 (40.0)	5 (33.33)	
Age, years, mean (SD)	8.30 (1.42)	8.60 (2.06)	1.0000
**ADHD subtype, N (%)**			
Predominantly IA type	8		
Predominantly HI type	0		
Combined	2		
Full-scale IQ, mean (SD)	101.60 (24.76)	105.93 (11.75)	0.3820
**Visual CPT scores, mean (SD)**			
Omission error	99.00 (9.23)	102.00 (7.27)	0.4120
Commission error	105.20 (16.46)	111.40 (13.09)	0.3272
Response time	74.50 (5.04)	83.27 (10.33)	0.0278*
Response time standard deviation	76.10 (9.28)	93.80 (16.28)	0.0138*
**Auditory CPT scores, mean (SD)**			
Omission error	100.70 (12.50)	103.20 (12.04)	0.4117
Commission error	97.50 (20.50)	108.00 (8.66)	0.2543
Response time	77.70 (9.90)	77.53 (8.42)	0.9536
Response time standard deviation	86.40 (14.77)	101.00 (15.54)	0.0480*
**ARS scores, mean (SD)**			
IA	11.00 (6.78)	5.47 (5.01)	0.0475*
HI	6.60 (5.99)	3.67 (4.62)	0.1909
Total	17.60 (11.36)	9.13 (9.43)	0.0668
**QAR scores, mean (SD)**			
Total	6615.60 (5046.45)	3404.20 (1716.51)	0.1546

*Indicates statistical significance at α = 0.05.

*ADHD* attention-deficit/hyperactivity disorder, *SD* standard deviation, *IA* inattention, *HI* hyperactivity-impulsivity, *IQ* intelligence quotient, *CPT* continuous performance test, *ARS* ADHD rating scale, *QAR* quantified assessment of hyperactivity using IR-UWB radar.

**Figure 1:**
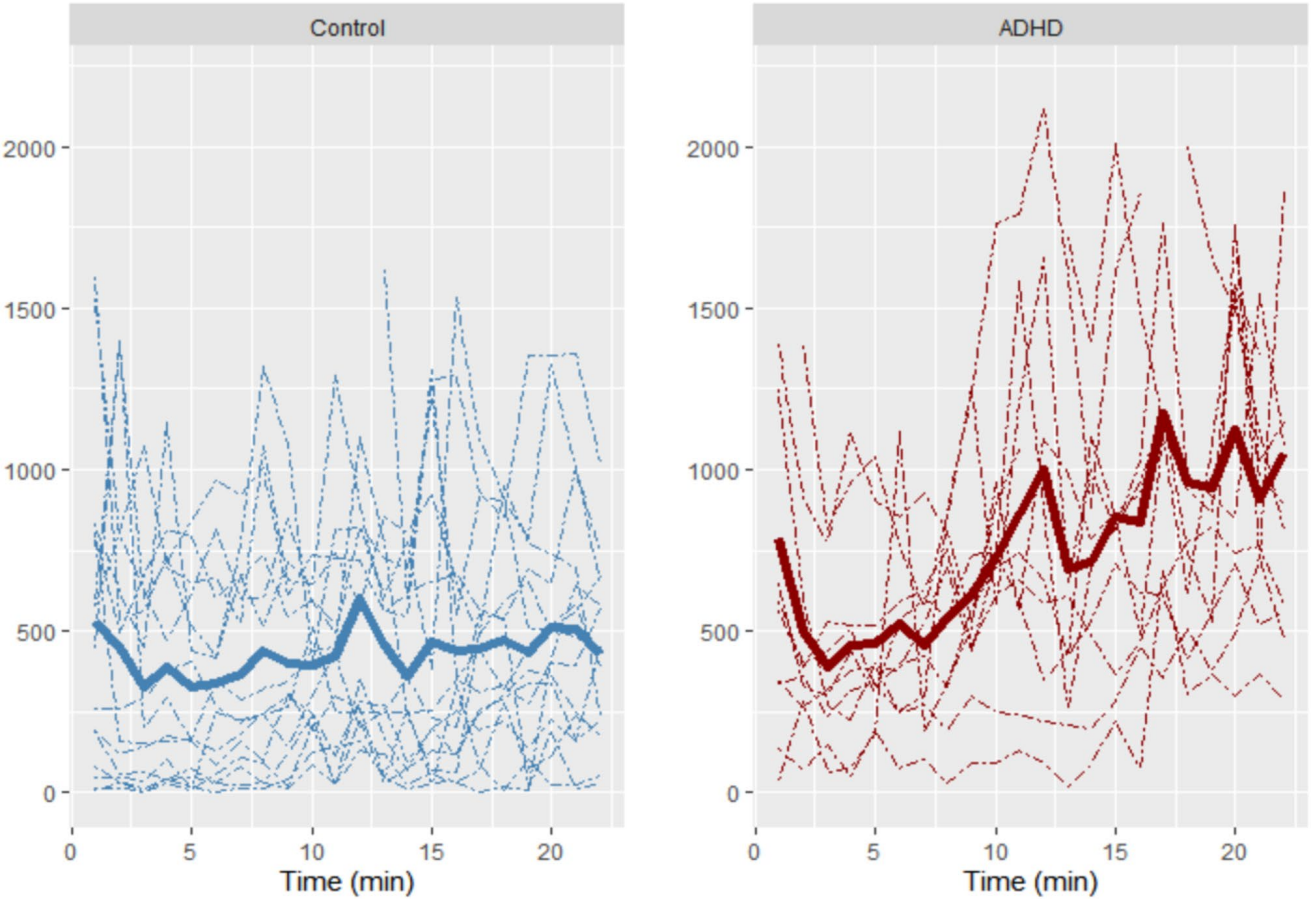
Total movements of the two groups during the CPT. *The dashed lines represent individual movements, while the bold, solid lines represent the sample mean functions. Abbreviations: ADHD, attention-deficit/hyperactivity disorder; CPT, continuous performance test.

**Figure 2:**
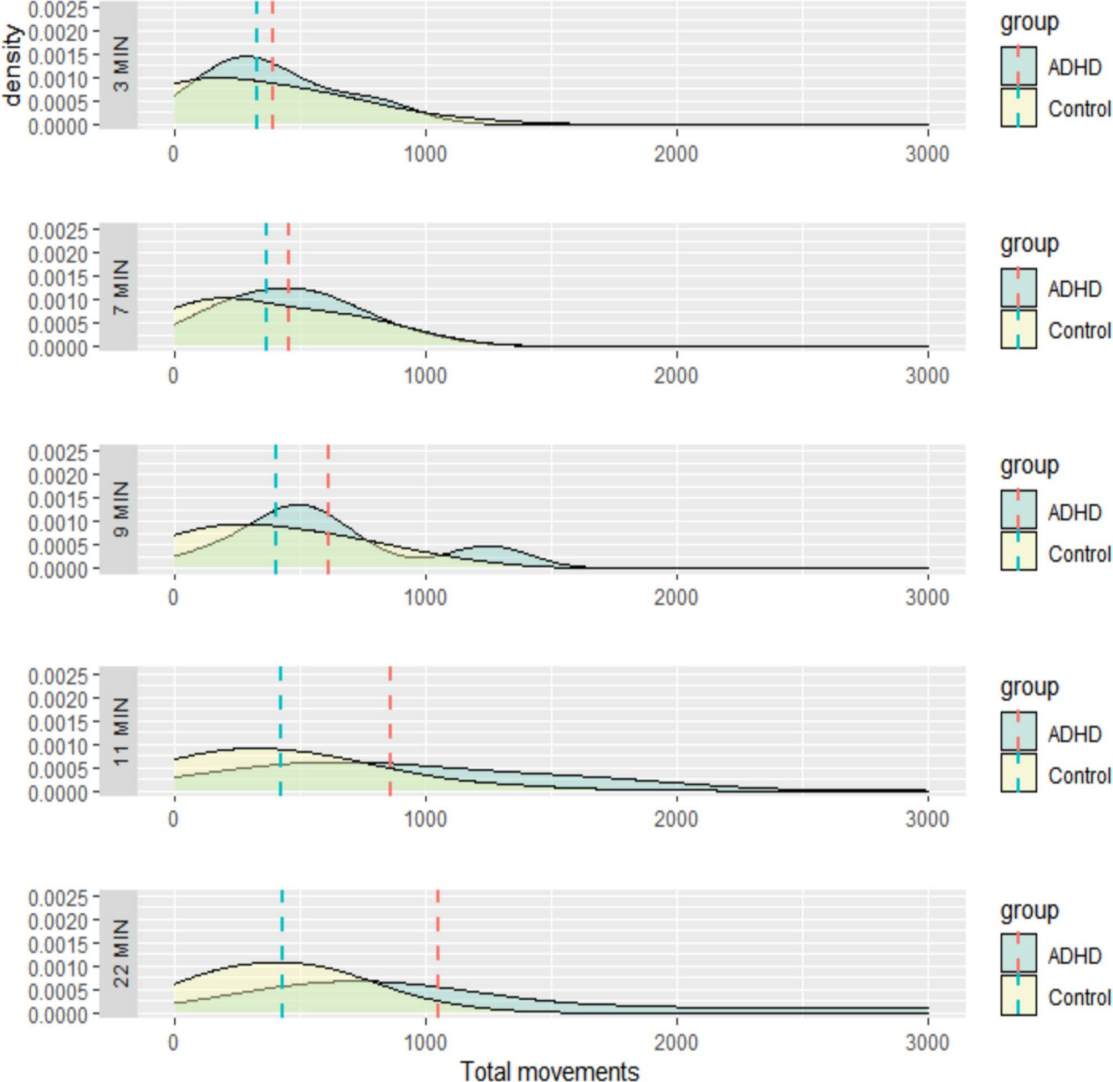
Distributional differences of the total movements at 3, 7, 9, 11 and 22 min. *The dashed vertical lines are point-wise means of the groups at each time. The sample means of the total movements for the normal control group stay almost the same, while those for ADHD group slightly increase over time, with high dispersion after approximately 7 min during the test.

The functional analysis of variance results, which compared the temporal variance in activity between the two groups, are summarized in Table 2. The mean functions for the movements during the test were significantly different between the ADHD and HC groups at the α = 0.05 level, while the velocities of the movement were not significantly different between the two groups. We also applied fANOVA to the visual and auditory CPTs separately and found that the differences in the mean functions of total movement were significant for only the auditory test (Table 3). The plot showing the pattern of total movements of the two groups indicates that while the movement of the HCs remained relatively consistent over time, the ADHD group showed a trend of increased movement over time (Fig. 1). However, the velocity of the total movements was stable over time in both groups (Supplementary Fig. S1). The sample means of the total movements for the HCs remained almost the same, while those for the ADHD group slightly increased over time with high dispersion after approximately 7 min into the test, which indicates that the difference in movement between the two groups became highly evident (Fig. 2). The total activity level in the ADHD group showed no significant correlation with the type of CPT, IQ, or ARS scores (Supplementary Table S1).

**Table 2:** fANOVA results over 22 min.

Category	Methods	Test statistics	*p*-values
Movements	Globalized pointwise F-test	3.9468	0.016*
	F-max bootstra*p* test	10.5414	0.029*
Movement velocity	Globalized pointwise F-test	0.9961	0.525
	F-max bootstra*p* test	5.0372	0.388

*Indicates statistical significance at α = 0.05, and bootstrapping was conducted 10,000 times.

*fANOVA* functional analysis of variance.

**Table 3:** fANOVA tests for the visual and auditory CPTs.

Test type	Methods	Test statistics	*p*-values
Visual	Globalized pointwise F-test	1.8376	0.183
	F-max bootstra*p* test	6.3363	0.086
Auditory	Globalized pointwise F-test	6.0560	0.002*
	F-max bootstra*p* test	10.5414	0.018*

*Indicates statistical significance at α = 0.05, and bootstrapping was conducted 10,000 times.

*fANOVA* functional analysis of variance, *CPT* continuous performance test.

## Discussion

This is the first study to use IR-UWB radar for the quantification of activity in ADHD patients and to apply fANOVA to investigate the temporal variance in activity levels during a CPT. Although there were no differences in the total activity level between the 2 groups, the patterns of activity level across time were useful in discriminating the ADHD and HC groups. When we separately investigated the activity levels across time according to task, only the results of the auditory task reached significance. This may be due to the order (thus the time effect) of the tasks, as the auditory tasks was always conducted after the visual task. The null results could be due to the small sample size, so further studies are warranted, but currently, our study results implicate that the temporal variance in activity could be more sensitive than total movement in hyperactivity assessment. Furthermore, the increase in activity in ADHD patients was evident in only a short period of time (7 min). The most frequently used parameters include movement frequency, intensity, duration, and distance, but no studies have considered the effect of time^6^. Further studies using indices reflecting the temporal variance in activity are warranted.

The temporal variance in activity levels in ADHD patients has been underinvestigated. According to previous studies, ADHD patients show significant decreases in activity and performance scores over time^19^. An actigraphy study also found a significant group x time effect on activity when testing activity levels over 2 days^5^. Another study analyzed the 24-h activity circadian rhythm by dividing the timeline into 1-h intervals^20^. A previous study found a significant group x time effect on activity during the Qb test when dividing the whole test period into 3 phases and calculating the parameters separately according to phase^17^. Hyperactivity was more prominent towards the end of the task; the motor activity of the ADHD patients was 3.5 times higher than that observed in the HCs. However, previous studies have been methodologically limited with regard to statistical analyses, as they analyzed the variance in mean activity across a certain interval of time (e.g., 5 min, 1 day, etc.). The activity level of participants should be treated as functional data, which are represented by functions or curves that reflect observations of a random variable over a continuous interval or in large discretizations. We suggest that future studies on movement or activity levels should implement fANOVA methods.

Our small sample size prohibited further analyses on the effect of sex, age and ADHD subtype on hyperactivity. Studies have consistently reported that boys exhibit more hyperactivity than girls^21^, but the results of the effect of age and ADHD subtype have been inconsistent. Some studies reported obvious hyperactivity decline from childhood to adulthood^22^, whereas other studies found no difference in the activity level between children and adults^6^. In contrast to the prevailing DSM-5 clinical view, some studies suggest that hyperactivity may be a cross-subtype and relatively homogeneous feature of ADHD despite clear differences in subjective perceptions regarding its presence/severity^6^. Although the majority of our ADHD population was predominantly inattentive, temporal variance in activity still differentiated the ADHD and HC groups, thus providing supporting evidence for these previous assumptions.

Our study was conducted during a CPT, but these results may vary according to test situations, as a meta-analysis by Kofler et al.^6^ suggested that the presence and magnitude of hyperactive behavior is context-dependent. Hyperactivity becomes prominent during high cognitive load, under high executive functioning demands and in low-stimulation environments^6^. Given the high within- and between-day variability in the classroom behavior of children with ADHD^23,24^, hyperactivity differences are likely to become increasingly large over a prolonged period^6^. Therefore, hyperactivity during a CPT and hyperactivity during other cognitive tests or in every-day situations may differ. This suggestion is supported by the minimal correlation between hyperactivity measured by the IR-UWB radar during the CPT task and the parent-rated hyperactivity level measured by questionnaires in our study. IR-UWB radar measured hyperactivity during a short CPT, which reflects a very specific moment in a laboratory setting, whereas parent-rated hyperactivity reflects the accumulation of behavior. Further studies in naturalistic settings, such as in the classroom, could be conducted to explore the nature of hyperactivity in real-life situations.

A strong advantage of IR-UWB radar is that it is a no contact method, causes minimal discomfort and is small and unnoticeable. It can be placed in various naturalistic settings to continuously monitor movement for long periods of time. Therefore, it could be used to collect data from normal-activity children, which is needed to establish a baseline for activity according to age and sex. IR-UWB radar may be used as a screening and treatment monitoring device for ADHD patients not only in the hospital but also in the classrooms and in the home.

### Limitations

This study has some notable limitations. First, the small sample size limited the study power. Comorbidity with tic disorders was not considered. There is a possibility that the table or chair used in the study restricted the participants’ movement or affected the detection of signals of the sensors. We minimized these confounding effects by using four radars from four directions and also used the median, rather than the mean, value of the signal detected from the four radars to quantify whole body movement. Future studies are warranted to determine the degree of signal reduction caused by surrounding objects. The concurrent validity was not examined; therefore, future studies should compare the results of IR-UWB radar and other devices, such as actigraphy or the Qb test. Finally, the majority of our ADHD patients were already taking medication; therefore, future drug-naïve or recent-onset studies are required.

## Methods

### Participants

A total of 10 young individuals with ADHD and 15 HCs were recruited from the Hanyang University Medical Center psychiatry outpatient clinic and the Seongdong-gu Community Mental Health Center from September 2019 to May 2020. The ADHD participants who were taking ADHD medication underwent assessment after a 1-week wash-out period. ADHD and other psychiatric comorbidities were confirmed according to the Diagnostic and Statistical Manual of Mental Disorders, Fourth Edition (DSM-IV) criteria by a board-certified child and adolescent psychiatrist using the Korean Kiddie Schedule for Affective Disorders and Schizophrenia—Present and Lifetime version (K-SADS-PL)^25^. The exclusion criteria for ADHD were as follows: an IQ < 70; a hereditary genetic disorder; a current or past history of brain trauma, an organic brain disorder, a seizure or any other neurological disorder; autism spectrum disorder, communication disorder or learning disorder; schizophrenia or any other childhood-onset psychotic disorder; major depressive disorder or bipolar disorder; Tourette’s syndrome or a chronic motor/vocal tic disorder; and obsessive compulsive disorder. The HC group was defined as community-dwelling children free of any psychiatric diagnosis according to the K-SADS-PL interview. The exclusion criteria for the HC group were the same as those for the ADHD group, with the addition of a diagnosis of ADHD.

This study was approved by the institutional review board of Hanyang University Medical Center (No. 2017-09-046-002). All methods were carried out in accordance with standard human research ethics guidelines (Declaration of Helsinki) and regulations. Written informed consent was obtained from the parents of the participants.

### Clinical assessment

The level of intelligence was assessed using the Wechsler Intelligence Scale for Children IV (WISC-IV)^26^. Full-scale IQ (FSIQ) scores were normalized to a mean of 100 and standard deviation of 15 based on Korean population-based reference data^27^. Higher scores indicate better cognitive performance.

ADHD symptoms were assessed by using a parent-report questionnaire called the ADHD Rating Scale IV (ARS)^28^. It consists of 18 items rated on a scale from 0–3, with potential responses of “never or rarely”, “sometimes”, “often”, and “very often”. The total score ranges from 0–54, with 9 items reflecting IA symptoms (IA scores range from 0–27) and 9 items rating hyperactivity-impulsivity (HI) symptoms (HI scores range from 0–27). Higher scores indicate greater severity.

A Korean version of a computerized CPT called the comprehensive attention test, which has established reliability and validity, was administered^29^. We used the visual selective attention task and auditory selective attention task, which are each 11 min long. Performance was assessed considering four variables: (1) omission errors (failure to respond; measurement of IA), (2) commission errors (faulty response; measurement of impulsivity), (3) response time (mean time of correct responses; measurement of processing speed), and (4) response time standard deviation (standard deviation of response time for correct responses; measurement of response time variability). All scores were transformed into attention quotients (AQs), which were adjusted for age and sex by comparison with a normal population with an average AQ of 100 and standard deviation of 15.

**Figure 3:**
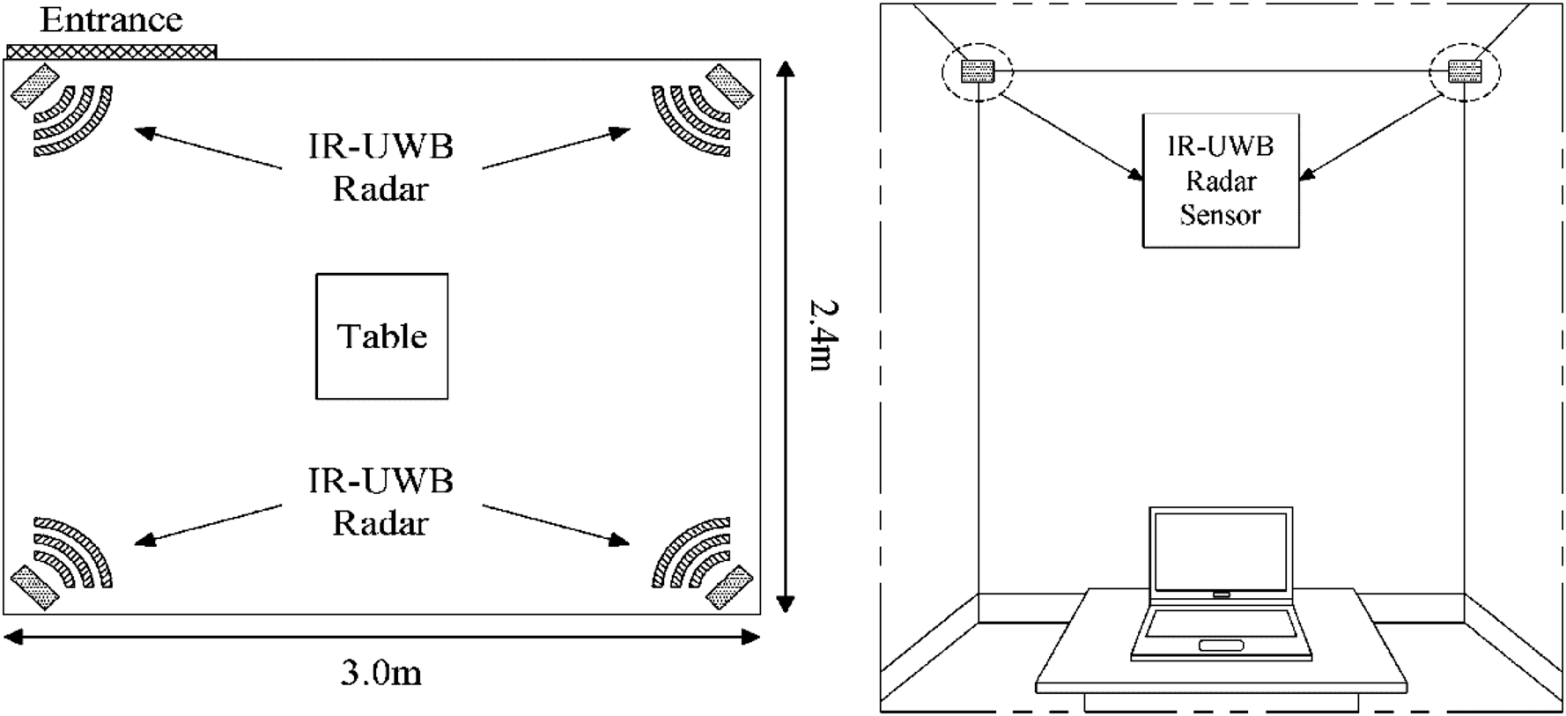
The IR-UWB radar hyperactivity assessment environment. IR-UWB, impulse-radio ultra-wideband.

Hyperactivity assessment was conducted in a 3.0 × 2.4 × 2.4 m wide space inside Hanyang University Hospital’s psychiatry outpatient clinic (Fig. 3). Age-appropriate tables and chairs, as well as laptops for the CPT, were placed in the middle of the room. The subjects entered the room alone and performed the CPT for a total of 22 min. Four IR-UWB radar sensors were installed at each corner on the ceiling. The four radars were connected via USB to a separate small PC. These sensors simultaneously monitored the participants during the 22-min CPT.

### Radar data collection and processing

IR-UWB radar sensor XK340 (Xandar Kardian, Toronto, ON, Canada) was used to quantify hyperactivity. XK-340 sensors can utilize a variety of center frequencies, but 8.748 GHz was chosen according to local regulations. The bandwidth was -10 db at approximately 1.5 GHz, and there was no interference between each sensor and between all multiple IR-UWB radar sensors. The radar sampled the received signals at 23.328 GS/s, and the sampled signals were transmitted to a PC in sequence via the USB interface. The radar's radiation power was 68.85 µW, which is lower than the radiation power used by communications such as Wi-Fi and Bluetooth. Each sensor was sealed inside a white plastic case to avoid attracting the attention of participants as much as possible. Due to the relatively small indoor environment, the sensors were installed radially because the volume of the human body could cause errors in distance or detection parameters. As other people's movements can interfere with those of the observed subjects during the test, the subjects were left alone during the test. Signals received from the sensors were loaded as digital values in MATLAB 2020a to run the signal processing algorithm. The PC received 20 signals per second from each sensor, and the PC's operating system was Windows 10.

### Signal model and basic signal processing

The impulse signal $s\left[ k \right]$ emitted from IR-UWB radar is reflected from the target being observed and from the surrounding environment. Reflected impulse signals received by the radar include noise components $N\left[ k \right]$ and multipass components in the surrounding environment.

1 $$x_{i} \left[ k \right] = \mathop \sum \limits_{m = 1}^{{N_{path} }} a_{m,i} s\left[ {k - \tau_{m,i} } \right] + N\left[ k \right]$$

The sampled time index $k$ can be considered a distance index and is expressed from 0 to $L_{signal}$, the maximum distance to be observed. $x_{i} \left[ k \right]$ is the signal received from the $m$-th path of the impulse signal and the $i$-th sensor. $a_{m,i}$ and $\tau_{m,i}$ are the scale value and delay value, respectively, and are determined by the surrounding environment^30^. Since radar sensors were installed indoors, *x* comprises a target signal as well as a background signal generated from a wall or static object. As these background signals have a large amplitudes and interfere with the target signal, it is necessary to remove the background signals so that only human motion can be extracted and observed. A background removal algorithm was used to observe only the moving target in the indoor environment^31^. The signal $y_{i,n} \left[ k \right]$ is the signal produced after static clutter $C_{i,n} \left[ k \right]$ is removed from the received $x_{i,n} \left[ k \right]$. Because the background removal algorithm is an algorithm for observing only moving objects, it plays a similar role to a high-pass filter. The background clutter signal $C_{i,n} \left[ k \right]$ is updated from the previous clutter signal $C_{i,n - 1} \left[ k \right]$ each time a signal is received, and n is the sequence of received signals. The background subtraction signal $y_{i,n} \left[ k \right]$ is expressed as follows:

2 $$y_{i,n} \left[ k \right] = x_{i,n} \left[ k \right] - C_{i,n} \left[ k \right],C_{i,n} \left[ k \right] = \alpha C_{i,n - 1} \left[ k \right] + \left( {1 - \alpha } \right)x_{i,n} \left[ k \right]$$

### Quantified assessment of hyperactivity

If there is no movement by the target indoors, the coefficient of the radar signal reflected and returned will not change. Conversely, if the target moves, the received signal x value will change as the electromagnetic wave path changes. Additionally, if the target moves, $x_{i,n} \left[ k \right]$ will change substantially because the distance from the radar to the target also changes. The distance resolution of IR-UWB radar is several millimeters, so it is possible to detect even small movements of the human body. Therefore, it is possible to quantify the degree of movement of a person through the amplitude of the received radar signal and the changing distance information. However, as the radar collects only one-dimensional data, it may be difficult to measure accurate movement because a body part near the sensor obscures a body part far away from the sensor. Therefore, four sensors were installed to quantify the movements so that the movements of the body could be measured in all directions.

Since the radar emits impulse signals $s\left[ k \right]$ at a high-frequency band of 7.6 GHz, it is difficult to quantify hyperactivity immediately using $y_{i,n} \left[ k \right]$. Accordingly, to analyze the movement of the subject, the analytic signal $z_{i,n} \left[ k \right]$ could be calculated using the Hilbert transform of $y_{i,n} \left[ k \right]$^32^. Then, the envelope of $y_{i,n} \left[ k \right]$ can be expressed as follows:

3 $$A_{i,n} \left[ k \right] = \left| {z_{i,n} \left[ k \right]} \right| = \left| {y_{i,n} \left[ k \right] + j\hat{y}_{i,n} \left[ k \right]} \right|$$

where $\hat{y}_{i,n} \left[ k \right]$ is the Hilbert transform of $y_{i,n} \left[ k \right]$, calculated as follows:

4 $$\begin{aligned} \hat{y}_{i,n} \left[ k \right] & = H\left[ {y_{i,n} \left[ k \right]} \right] = y_{i,n} \left[ k \right]*DFT^{ - 1} \left[ { - i sgn\left[ k \right]} \right] \\ & = \left\{ {\begin{array}{*{20}c} {\frac{2}{\pi }\mathop \sum \limits_{l = odd} \frac{{y_{i,n} \left[ k \right]}}{k - l};\quad k is even} \\ {\frac{2}{\pi }\mathop \sum \limits_{l = even} \frac{{y_{i,n} \left[ k \right]}}{k - l};\quad k is odd} \\ \end{array} } \right. \\ \end{aligned}$$

Equation (2) is also called the moving target indication filter, so $A_{i,n} \left[ k \right]$ is proportional to the movement. Similar to that in Eq. (1), the received signal also contains noise $N\left[ k \right]$, which needs to be removed before quantifying the activity. This can be done using the threshold $T_{i}$ based on difference of consecutive radar signals collected in an empty environment where there are no people for a certain amount of time^33^. To quantify movement, the movement of each subject is detected by the received signal and the threshold, and it can be expressed as follows:

5 $$g_{i,n} \left[ k \right] = \left\{ {\begin{array}{*{20}l} 1 \hfill & {if \left| {A_{i,n} \left[ k \right] - A_{i,n - 1} \left[ k \right]} \right| \ge T_{i} } \hfill \\ 0 \hfill & {if \left| {A_{i,n} \left[ k \right] - A_{i,n - 1} \left[ k \right]} \right| < T_{i} } \hfill \\ \end{array} } \right.$$

$g_{i,n} \left[ k \right]$ is calculated as the difference of signals before and after Hilbert transformation. Previously, movement was represented by the difference in amplitude of two consecutive signals^16,34,35^. If the subject's movement is substantial, the difference in signals $A_{i,n} \left[ k \right]$ and $A_{i,n - 1} \left[ k \right]$ over time will also be substantial, so there will be a large number of values of $g_{i,n} \left[ k \right]$ that are determined to be 1 according to the signal created by the difference between $A_{i,n} \left[ k \right]$ and $A_{i,n - 1} \left[ k \right]$.

6 $$Q_{i} \left[ n \right] = \mathop \sum \limits_{k = 0}^{{L_{signal} }} g_{i,n} \left[ k \right]$$

7 $$QAR\left[ n \right] = Median\left( {Q_{1} \left[ n \right], Q_{2} \left[ n \right], Q_{3} \left[ n \right], Q_{4} \left[ n \right]} \right)$$

where $Q_{i} \left[ n \right]$ equals the number of $g_{i,n} \left[ k \right]$ components with a value of 1. When movement exists and there is a change, $n$-th $g_{i,n} \left[ k \right]$ will result in a large value of $Q_{i} \left[ n \right]$. This approach can quantify the subject's hyperactivity. Since a total of four sensors covered the subject's body from different directions, it is possible that certain body parts masked the movements of other body parts, and the radar cross section (RCS) can be measured differently, even with the same movement. The median of the $Q_{i} \left[ n \right]$ values observed by the four sensors was considered $QAR\left[ n \right]$. This accounts for the aforementioned problems and objectively evaluate the hyperactivity of subjects. Since the radar's frames per second (FPS) value was 20, the above algorithm calculates 20 $QAR$ values per second. To analyze movement over time, the obtained $QAR$ values are statistically computed by averaging the values in approximately one minute. As a result, 22 $QAR$ values are calculated for one subject.

### Statistical analysis

The demographic and clinical characteristics of the 2 groups were compared using the Wilcoxon rank sum test for continuous variables and chi-square test for categorical variables. The study tested the difference in the mean functions between the two groups using one-way fANOVA under the null hypothesis of $H_{0} : \mu_{1} \left( t \right) = \mu_{2} \left( t \right)$ at α = 0.05 to compare the total movements performed during the 22-min visual and auditory CPT between the ADHD and HC groups. The mean functions were constructed from the random functions over the discretized time interval of [0, 22], and fANOVA was performed based on the globalized pointwise F-test^36^ and F-max test, with 1,000 bootstrap replications^37^, which maintained the preassigned α-level^26^. We also tested whether the velocities of the total movements between the two groups were significantly different over time; the velocity was defined as the change rate of the total movements per minute. In addition, we also observed the sample distributional differences between the two groups at 3, 7, 9, 11 and 22 min by comparing the density distributions. We nonparametrically examined the correlations between selective attention tests and total movements based on Spearman’s correlation coefficient, with statistical significance of the correlation coefficients considered at α = 0.05. The total movement was quantified as a single value for each subject by approximately integrating the area under the functional curve through the end of the test time. All of the statistical analyses and figure generations were performed using R software (version 3.6.0; https://cran.r-project.org/).

## Conclusions

We found that IR-UWB radar can be useful in discriminating the mean functions of activity level during a CPT in ADHD and HC groups when applying fANOVA as a statistical method. Further studies with larger sample sizes are warranted to elucidate the effects of age, sex, subtype and situation.

## Supplementary Information

Supplementary Information.

## Data Availability

Data may be available upon request.
