# Supplementary material for: Quantified assessment of hyperactivity in ADHD youth using IR-UWB radar
Source: Sci Rep. 2021 May 5;11:9604. doi: 10.1038/s41598-021-89024-7 (PMC8100299; doi:10.1038/s41598-021-89024-7)
Supplement: Supplementary file 1 — Supplementary Information. [file 41598_2021_89024_MOESM1_ESM.docx]

**Quantified Assessment of Hyperactivity in ADHD Youth Using IR-UWB Radar**

Won Hyuk Lee,^1^† Johanna Inhyang Kim,^2^† Amy M. Kwon,^3^ Jong Ho Cha,^4^ Daehyeon Yim,^5^ Young-Hyo Lim,^6^ Seok-Hyun Cho,^7^ Sung Ho Cho,^1^* Hyun-Kyung Park^4^*

^1^Department of Electronics and Computer Engineering, Hanyang University, Seoul, Republic of Korea

^2^Department of Psychiatry, Hanyang University Medical Center, Seoul, Republic of Korea

^3^Biostatistical Consulting and Research Laboratory, Medical Research Collaborating Center, Seoul, Republic of Korea

^4^Department of Pediatrics, Hanyang University College of Medicine, Seoul, Republic of Korea

^5^Xandar Kardian Inc, Seoul, Republic of Korea

^6^Division of Cardiology, Department of Internal Medicine, Hanyang University College of Medicine, Seoul, Republic of Korea

^7^Department of Otorhinolaryngology, Hanyang University College of Medicine, Seoul, Republic of Korea

†Won Hyuk Lee and Johanna Inhyang Kim contributed equally.

**Corresponding Author**

*Hyun-Kyung Park, MD, PhD

Division of Neonatology, Department of Pediatrics, Hanyang University College of Medicine, 222 Wangsimni-ro, Seongdong-gu, Seoul 04763, Republic of Korea

Tel.: +82-2-2290-8397/+82-10-4749-1454, Fax: +82-2-2297-2380

Email: neopark@hanyang.ac.kr

*Sung Ho Cho, PhD

Department of Electronics and Computer Engineering, Hanyang University, 222 Wangsimni-ro, Sungdong-gu, Seoul 04763, Republic of Korea.

Tel.: +82-2-2220-0390/+82-10-5412-5178, Fax: +82-2-2220-4883

Email: dragon@hanyang.ac.kr

**Supplementary Figures**


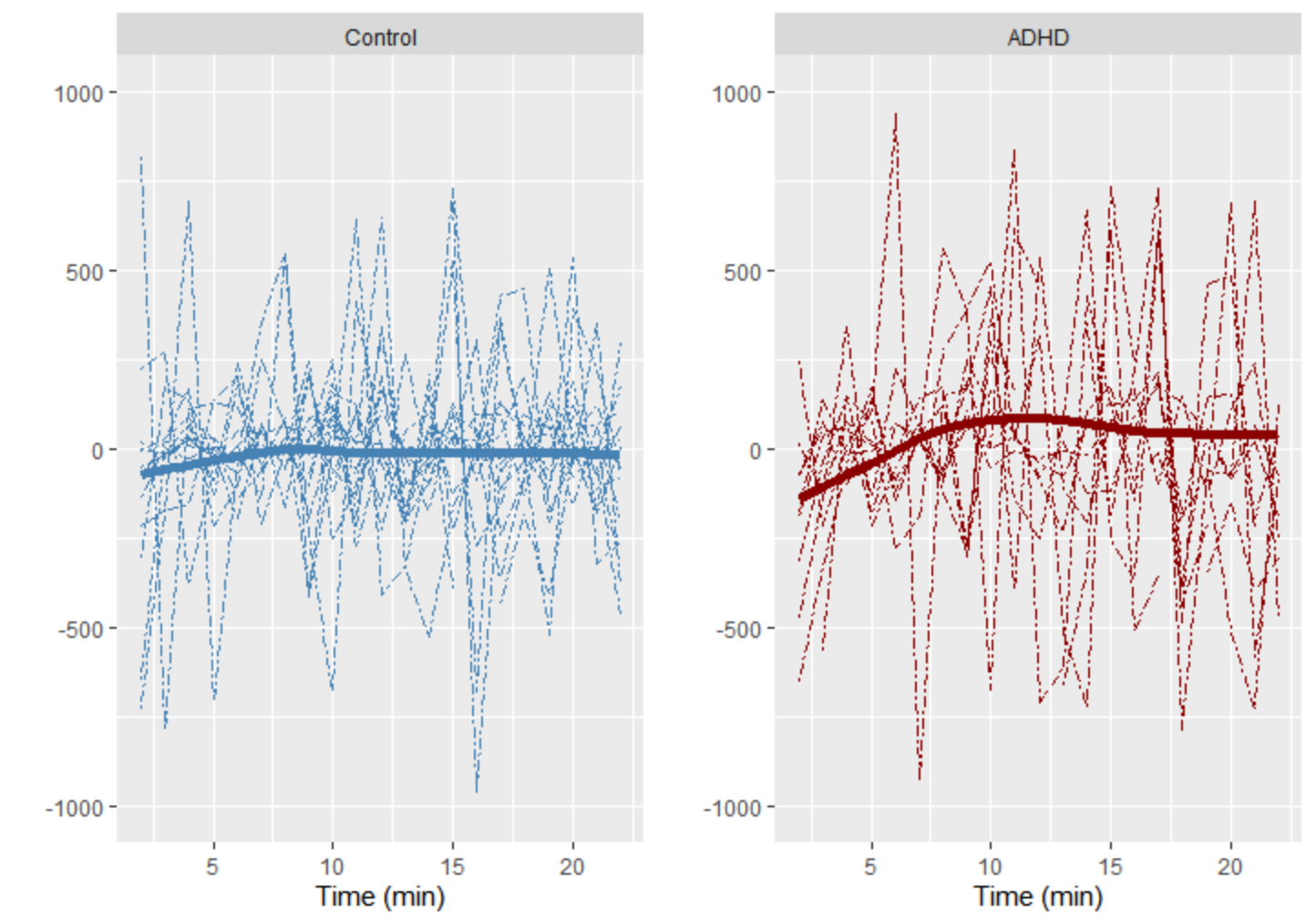


**Supplementary Fig. S1. Velocities of total movements over time.** *The dashed lines represent an individual’s velocity of total movement, while the bold, solid lines represent locally weighted scatter plot smoothing of the mean velocities of the total movements.

**Supplementary Tables**

**Supplementary Table S1.** Correlations between selective attention and total movement by group.

|  |  | ADHD | HCs |
| --- | --- | --- | --- |
| Visual selective attention | |  |  |
|  | Omission error | 0.48 (0.16) | -0.16 (0.57) |
|  | Commission error | 0.45 (0.19) | -0.40 (0.14) |
|  | Response time | -0.19 (0.59) | -0.04 (0.88) |
|  | Response time (SD) | 0.30 (0.40) | -0.18 (0.51) |
| Auditory selective attention | |  |  |
|  | Omission error | 0.18 (0.62) | 0.05 (0.86) |
|  | Commission error | 0.21 (0.56) | 0.03 (0.91) |
|  | Response time | -0.16 (0.66) | -0.28 (0.31) |
|  | Response time (SD) | 0.21 (0.57) | 0.02 (0.94) |
| Full-scale IQ | | -0.02 (0.96) | 0.19 (0.56) |
| ARS scores | |  |  |
|  | IA | -0.48 (0.22) | 0.34 (0.28) |
|  | HI | 0.08 (0.84) | 0.39 (0.21) |
|  | Total | -0.24 (0.57) | 0.36 (0.25) |

† The value in the first row in each cell represents Spearman’s correlation coefficient, and the value in the second row shows the corresponding p-value of the correlation coefficient.

Abbreviations: ADHD, attention-deficit/hyperactivity disorder; HC, healthy control; IQ, intelligence quotient; SD, standard deviation; ARS: ADHD rating scale; IA, inattention score; HI, hyperactivity-impulsivity score
